# L‐Theanine Suppresses the Increase in Blood Glucose Levels in Mice During Glucose Tolerance Tests by Promoting Glucose Excretion in the Urine

**DOI:** 10.1002/fsn3.70996

**Published:** 2025-10-09

**Authors:** Shinnosuke Yamaura, Koki Sadamori, Koichi Kawada, Kyosuke Uno, Reiko Konishi, Takashi Majima, Akira Mukai, Koji Komori, Nobuyuki Kuramoto, Kou Kawada

**Affiliations:** ^1^ Laboratory of Clinical Pharmacology and Therapeutics, Faculty of Pharmaceutical Sciences Setsunan University Hirakata, Osaka Japan; ^2^ Department of Bioactive Molecules, Pharmacology Gifu Pharmaceutical University Gifu Japan; ^3^ Laboratory of Molecular Pharmacology, Faculty of Pharmaceutical Sciences Setsunan University Hirakata, Osaka Japan; ^4^ Faculty of Sports and Health Science, Department of Nursing Daito Bunka University Higashimatsuyama, Saitama Japan

**Keywords:** amino acid transporter, antidiabetic effects, blood glucose level, glucose tolerance test, l‐theanine, urinary glucose

## Abstract

L‐theanine (N‐ethyl‐γ‐glutamine), a major amino acid in tea leaves, has been reported to exert anti‐obesity and antidiabetic effects; however, its mechanisms remain unclear. This study aimed to examine the impact of L‐theanine on blood glucose levels during an oral glucose tolerance test (OGTT) in mice, along with its effects on urinary glucose and amino acid excretion. Male Std‐ddY mice (5–8 weeks old) received oral L‐theanine at 100 or 1000 mg/kg 15 min before OGTT. Blood glucose levels were measured using a blood glucose meter 15 min prior to OGTT and at 0, 15, 30, 60, and 120 min post‐OGTT. Urinary glucose concentrations were measured using the electrode method after oral administration of L‐theanine for 15 min until the start of OGTT and every 30 min for 180 min after the start of OGTT. Concentrations of urinary amino acids, including L‐theanine, were quantified using the PTC‐HPLC method. Mice administered 1000 mg/kg L‐theanine showed significantly lower blood glucose levels at 15, 30, and 60 min postglucose loading compared to controls. Urinary glucose concentration was also significantly higher. Additionally, the concentration of glutamic acid, glutamine, leucine, serine, methionine, glycine, valine, and lysine increased significantly. These results suggest that L‐theanine reduces postprandial blood glucose by promoting urinary glucose excretion. It may interfere with renal glucose reabsorption via competition at amino acid transporters. L‐theanine could therefore be a useful compound for managing blood glucose levels.

## Graphical abstract

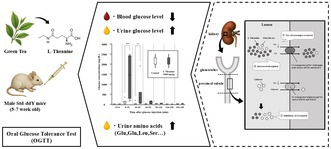

## 1 Introduction

The growing population with obesity and hyperglycemia is a major problem in recent society. Preventing obesity and hyperglycemia is important for reducing the risk of developing diabetes. In recent years, several foods for specified health uses and supplements aimed at improving obesity and hyperglycemia have been marketed. L‐theanine, an amino acid contained in green tea, is a neutral amino acid with a structure similar to L‐glutamine and is known as the umami component of tea (Sharma et al. 2018; Yoneda et al. 2020). L‐theanine is known as a dietary supplement that can provide relaxing and sleep‐improving effects, and in recent years, its potential for anti‐obesity and antidiabetic effects has attracted attention (Adhikary and Mandal 2017; Li et al. 2022).

Many studies have reported that tea consumption is effective against obesity and diabetes in humans, but a short history of research links L‐theanine with diabetes (Brimson et al. 2022; Cheng et al. 2024; Kao et al. 2006). According to a clinical study conducted by Ninomiya et al. in 2009, an increase in the blood concentration of L‐theanine metabolites reduced the risk of developing type 2 diabetes by approximately 30% (Ninomiya et al. 2019). However, in this report, the blood levels of L‐theanine are unknown, and the mechanism by which it reduced the risk of developing type 2 diabetes is not described. A few studies in rats and mice clarify the mechanism of the anti‐obesity and antidiabetic effects of L‐theanine. Yan et al. reported that L‐theanine administration for 2 weeks in rats inhibited the expression of SGLT3 and GLUT5 mRNA in the intestinal tract, suppressed glucose absorption from the small intestine, and reduced serum glucose and insulin concentrations (Yan et al. 2017). Peng et al. also reported that treatment of mice with L‐theanine for 10 weeks activated AMPK in adipocytes, promoted Prdm16 expression, and changed from energy‐storing white adipose tissue to energy‐consuming brown adipose tissue, improving weight gain caused by high‐fat diet intake and insulin sensitivity to a high‐fat diet (Peng et al. 2021). As these reports suggest, it is becoming clear that L‐theanine has anti‐obesity and antidiabetic effects by affecting blood glucose levels and insulin secretion in rats and mice. However, although some partial reports aim at elucidating the mechanisms of effects on sugar absorption and metabolism, no reports clarify the direct effects or phenomena on blood glucose levels.

Therefore, we clarified whether L‐theanine intake suppresses the rise in blood glucose level and focused on its effect on urinary glucose excretion as one of the mechanisms. In this study, the effects of L‐theanine intake on blood glucose and blood insulin levels in mice during a glucose tolerance test (OGTT) were examined, and urinary amino acid concentrations were measured in addition to urinary glucose concentrations during the test. The transporters (e.g., SGLTs and ASCT2) that reabsorb glucose and amino acids excreted in urine are known to cotransport sodium ions (Na^+^) (Avissar et al. 2001; Pizzagalli et al. 2021; Scalise et al. 2018; Wright 2001; Wright et al. 2011). Therefore, in this study, we also measured urinary Na^+^ concentrations and evaluated their effects on glucose and amino acid reabsorption.

## 2 Materials and Methods

### 2.1 Experimental Reagents and Equipment

L‐Theanine was purchased from Taiyo Kagaku Co. Ltd. (Mie, Japan). All reagents for identifying amino acids in urine, including amino acid standard solution type H, were purchased from FUJIFILM Wako Pure Chemical Corporation (Osaka, Japan). The measuring device and chips used to measure the blood glucose levels in mice were purchased from Terumo Corporation (Tokyo, Japan). A kit for measuring blood insulin concentrations in mice was purchased from Morinaga Institute of Biochemistry Co. Ltd. (Kanagawa, Japan). The device for measuring urinary sodium concentration, LAQUA twin Na‐11, was purchased from HORIBA Ltd. (Kyoto, Japan). Urinary glucose concentrations were quantified using an electrode method by Oriental Yeast Co. Ltd. (Tokyo, Japan).

### 2.2 Animals and Ethical Considerations

Male Std‐ddY mice (5–7 weeks old, average body weight: 30.3 g) were purchased from SHIMIZU Laboratory Supplies Co. Ltd. (Kyoto, Japan). All mice were housed under controlled conditions (temperature: 22°C ± 2°C; humidity: 55% ± 5%; 12‐h light/dark cycle) with free access to food and water. This study was approved and conducted in accordance with the guidelines of Setsunan University by the Institutional Animal Care and Use Committee (approval numbers: K22‐27 and K23‐26).

### 2.3 OGTT and Measurement of Blood Glucose and Blood Insulin Levels

Mice were divided into control and treatment groups. The treatment group was further divided into two subgroups receiving L‐theanine at a dose of 100 or 1000 mg/kg. After a 6‐h fasting period, the control group was administered purified water orally, whereas the treatment group received L‐theanine via oral gavage. The OGTT was initiated 15 min after the administration of purified water or L‐theanine by orally administering a glucose solution at 2 g/kg body weight. Blood glucose levels were measured at −15 min (immediately after the administration of purified water or L‐theanine), 0 min (immediately after glucose administration), and at 15, 30, 60, and 120 min postglucose administration. Simultaneously, with blood glucose measurements, blood samples were collected for the determination of plasma insulin levels using a mouse/rat insulin ELISA kit.

### 2.4 Collection of Urine and Measurement of Urinary Glucose and Sodium Concentrations

Urine was collected using individual mouse metabolic cages purchased from TECNIPLAST Co. Ltd. (Tokyo, Japan). Specifically, urine was collected during the 15‐min period between the oral administration of purified water or L‐theanine and the start of the OGTT (−15 to 0 min), and then every 30 min following the initiation of the OGTT (0–30, 30–60, 60–90, 90–120, 120–150, and 150–180 min). Urine samples were collected from the metabolic cages and used to measure sodium ion concentrations using a sodium ion measurement device. Glucose concentrations were determined by Oriental Yeast Co. Ltd. using an electrode‐based method.

### 2.5 Amino Acid Analysis in Urine and Statistical Processing

L‐theanine and other amino acids in the urine samples obtained were analyzed and quantified using the PITC method according to our previously established and reported method (Yamaura et al. 2024). The values obtained in the experiment were statistically evaluated using the Wilcoxon rank sum test, and differences were considered significant when the significance level p was less than 0.05.

## 3 Results

### 3.1 Blood Glucose and Blood Insulin Levels During OGTT

**FIGURE 1:**
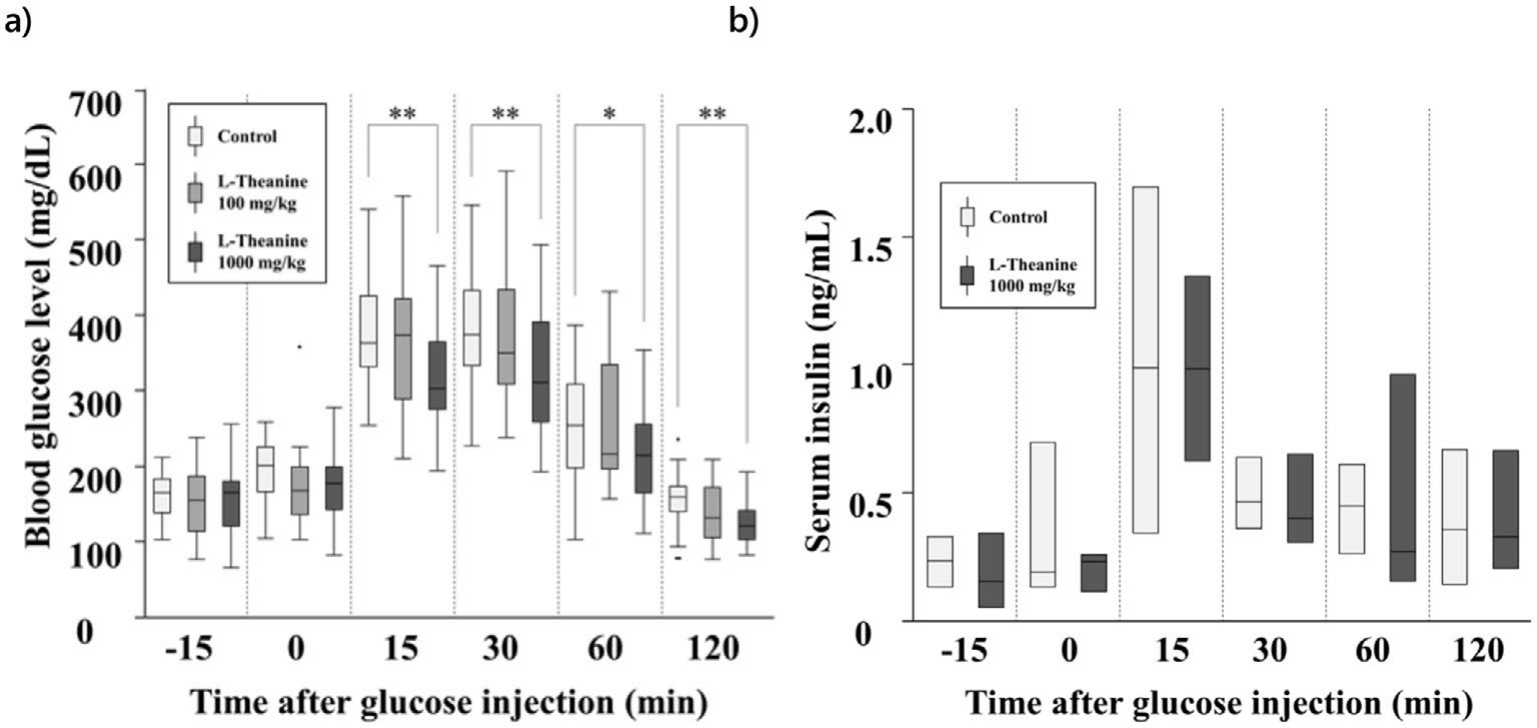
Blood glucose and insulin concentrations after administration of purified water or L‐theanine in OGTT. Values are displayed using box‐and‐whisker plots, wherein the box represents the interquartile range (25th–75th percentiles) and the median is indicated using a line within the box. Outliers beyond the typical range are plotted as individual dots. (a) For blood glucose levels (mg/dL), the sample sizes were as follows: Control group (*n* = 36), L‐theanine 100 mg/kg group (*n* = 17), and L‐theanine 1000 mg/kg group (*n* = 35). (b) For insulin concentrations (ng/mL), the sample sizes were: Control group (*n* = 3) and L‐theanine 1000 mg/kg group (*n* = 3). Statistically significant differences are indicated by asterisks (*p < 0.05, **p < 0.01).

The changes in blood glucose levels during the OGTT are shown in Figure 1a, and the corresponding insulin concentrations at the same time points are presented in Figure 1b. The numerical values of these results are provided in Table 1. Blood glucose levels in the L‐theanine 100 mg/kg group did not differ significantly from those in the control group. In contrast, the L‐theanine 1000 mg/kg group exhibited significantly lower blood glucose levels compared with the control group at 15, 30, 60, and 120 min. No significant differences in blood insulin levels were observed between the control group and the group treated with 1000 mg/kg L‐theanine. Both groups showed a transient increase to approximately 1.0 ng/mL at 15 min after glucose administration. The insulin concentration level decreased to approximately 0.4 ng/mL at 30 min after glucose administration and remained unchanged until 120 min afterward.

**TABLE 1:** Changes in blood glucose and insulin concentrations during the OGTT.

Group		−15	0	15	30	60	120
	Control	166.0 [139.3–184.0]	201.5 [166.3–225.5]	363.5 [331.8–425.8]	374.5 [334.3–432.5]	255.0 [199.0–309.5]	160.0 [140.3–173.8]
Blood glucose (mg/dL)	L‐Theanine 100 mg/kg	156.0 [114.5–187.5]	168.0 [136.0–200.5]	374.0 [289.0–422.5]	350.0 [308.5–434.0]	216.0 [196.5–335.0]	133.0 [106.0–172.5]
	L‐Theanine 1000 mg/kg	166.0 [121.0–181.0]	178.0 [144.0–200.0]	304.0 [276.0–365.0]	312.0 [259.0–392.0]	215.0 [166.0–257.0]	121.0 [103.0–142.0]
Insulin concentrations (ng/mL)	Control	0.24 [0.14–0.33]	0.19 [0.14–0.70]	0.99 [0.34–1.69]	0.47 [0.36–0.64]	0.45 [0.27–0.61]	0.36 [0.14–0.67]
	L‐Theanine 1000 mg/kg (*n* = 3)	0.16 [0.05–0.34]	0.23 [0.12–0.26]	0.98 [0.63–1.35]	0.40 [0.31–0.65]	0.27 [0.16–0.96]	0.33 [0.21–0.67]

*Note:* In the upper panel (blood glucose, mg/dL), the sample sizes were: control group (*n* = 36), L‐theanine 100 mg/kg group (*n* = 17), and L‐theanine 1000 mg/kg group (*n* = 35). In the lower panel (insulin concentrations, ng/mL), the sample sizes were: control group (*n* = 3) and L‐theanine 1000 mg/kg group (*n* = 3).

### 3.2 Amount of Urine Excreted During OGTT and Urinary Glucose Concentration

**FIGURE 2:**
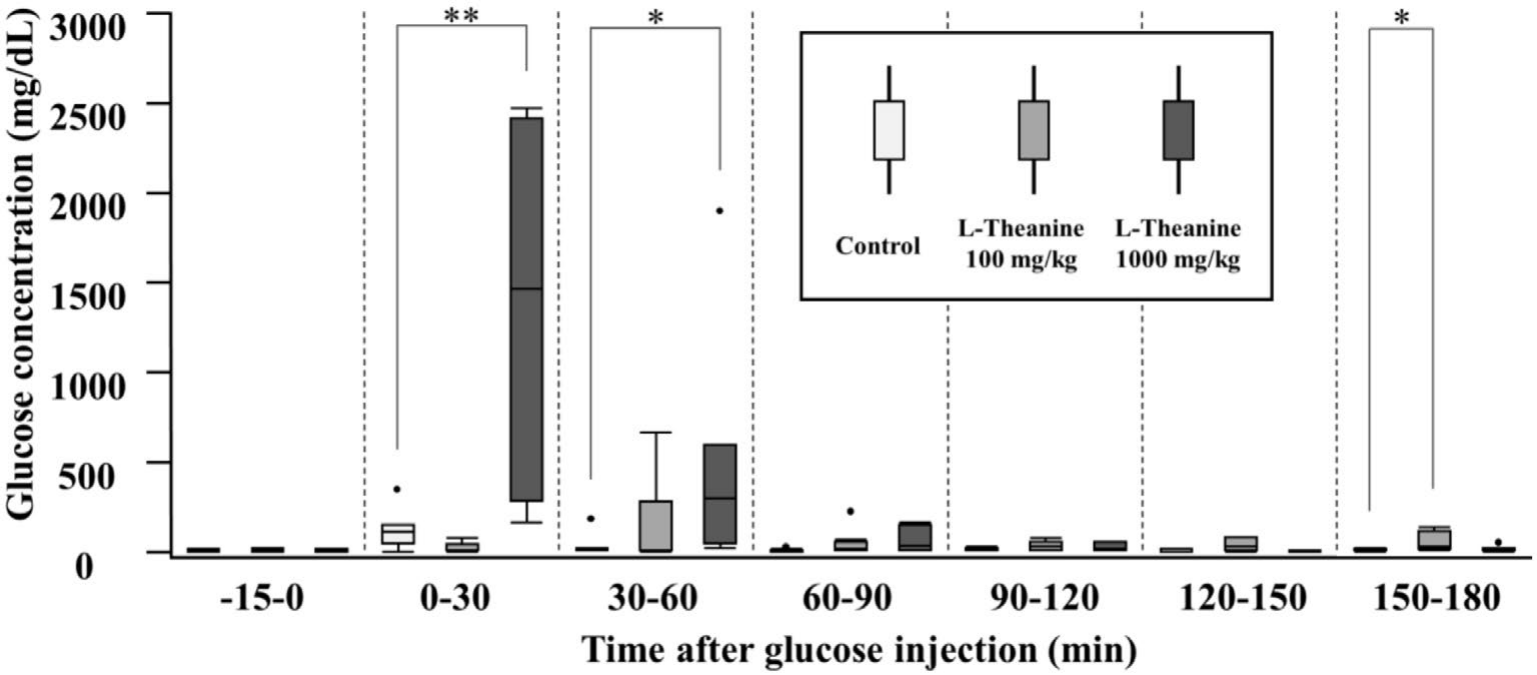
Urinary glucose concentrations during OGTT. Values are displayed using box‐and‐whisker plots, wherein the box represents the interquartile range (25th–75th percentiles) and the median is indicated using a line within the box. Outliers beyond the typical range are plotted as individual dots. Sample sizes for each group were as follows: Control group (−15–0 min: *N* = 5; 0–30 min: *N* = 7; 30–60 min: *N* = 8; 60–90 min: *N* = 8; 90–120 min: *N* = 5; 120–150 min: *N* = 5; 150–180 min: *N* = 6), L‐theanine 100 mg/kg group (−15–0 min: *N* = 4; 0–30 min: *N* = 6; 30–60 min: *N* = 10; 60–90 min: *N* = 8; 90–120 min: *N* = 5; 120–150 min: *N* = 5; 150–180 min: *N* = 5), and L‐theanine 1000 mg/kg group (−15–0 min: *N* = 3; 0–30 min: *N* = 4; 30–60 min: *N* = 7; 60–90 min: *N* = 6; 90–120 min: *N* = 3; 120–150 min: *N* = 4; 150–180 min: *N* = 7). Statistically significant differences are indicated by asterisks (*p < 0.05, **p < 0.01).

Urinary excretion volumes at each time point during the OGTT are presented in Table 2, and the corresponding urinary glucose concentrations are shown in Figure 2 and Table 3. No significant differences in urinary volume during the OGTT were observed between either the L‐theanine 1000 mg/kg or 100 mg/kg group and the control group. In all groups, urinary volume was low during the −15 to 0 min interval and tended to increase between 30 and 60 min. In the 1000 mg/kg L‐theanine group, urinary glucose concentrations were significantly higher during the 0–30 min and 30–60 min intervals than those in the control group. In the 100 mg/kg group, urinary glucose concentration peaked at 30–60 min; however, no significant difference was observed during other time periods compared with the control group. Additionally, the 100 mg/kg group showed a higher glucose concentration in urine during 150–180 min than the control group.

**TABLE 2:** Urine volume during the OGTT.

Group	−15–0	0–30	30–60	60–90	90–120	120–150	150–180
Control	81.6 [62.8–156.3]	191.1 [119.2–215.7]	325.2 [230.6–435.9]	228.1 [173.5–332.5]	234.7 [165.9–334.2]	183.6 [63.7–466.3]	224.5 [111.2–364.5]
L‐Theanine 100 mg/kg	88.3 [65.0–145.0]	264.1 [167.8–313.3]	359.0 [175.2–405.3]	359.3 [175.3–454.3]	187.2 [134.8–321.4]	272.5 [214.6–548.4]	240.9 [162.3–356.3]
L‐Theanine 1000 mg/kg	106.8 [37.0–144.5]	214.4 [138.9–315.9]	450.0 [214.2–516.5]	182.1 [135.3–478.3]	214.5 [85.4–219.4]	121.4 [83.1–159.2]	320.7 [149.2–421.2]

*Note:* Control group (−15–0 min: *n* = 5; 0–30 min: *n* = 7; 30–60 min: *n* = 8; 60–90 min: *n* = 8; 90–120 min: *n* = 5; 120–150 min: *n* = 5; 150–180 min: *n* = 6), L‐theanine 100 mg/kg group (−15–0 min: *n* = 4; 0–30 min: *n* = 6; 30–60 min: *n* = 10; 60–90 min: *n* = 8; 90–120 min: *n* = 5; 120–150 min: *n* = 5; 150–180 min: *n* = 5), and L‐theanine 1000 mg/kg group (−15–0 min: *n* = 3; 0–30 min: *n* = 4; 30–60 min: *n* = 7; 60–90 min: *n* = 6; 90–120 min: *n* = 3; 120–150 min: *n* = 4; 150–180 min: *n* = 7).

**TABLE 3:** Urinary glucose concentrations at each time point during the OGTT.

Group	−15‐0	0–30	30–60	60–90	90–120	120–150	150–180
Control	12.0 [6.0–14.0]	112.0 [52.0–151.0]	15.5 [10.3–22.5]	8.0 [6.3–12.8]	9.5 [8.0–25.3]	6.0 [3.0–14.0]	11.0 [8.5–15.8]
L‐Theanine 100 mg/kg	11.5 [3.3–21.3]	12.0 [7.8–46.8]	12.5 [8.3–283.5]	14.0 [9.0–57.8]	32.0 [9.0–56.0]	29.0 [8.0–87.5]	29.0 [15.0–121.5]
L‐Theanine 1000 mg/kg	11.0 [7.0–15.0]	1462.0 [286.5–2420.0]	302.0 [54.0–596.0]	38.0 [10.5–154.8]	17.0 [11.0–56.0]	9.0 [6.8–11.3]	13.0 [8.0–24.0]

*Note:* Control group (−15–0 min: *n* = 5; 0–30 min: *n* = 7; 30–60 min: *n* = 8; 60–90 min: *n* = 8; 90–120 min: *n* = 5; 120–150 min: *n* = 5; 150–180 min: *n* = 6), L‐theanine 100 mg/kg group (−15–0 min: *n* = 4; 0–30 min: *n* = 6; 30–60 min: *n* = 10; 60–90 min: *n* = 8; 90–120 min: *n* = 5; 120–150 min: *n* = 5; 150–180 min: *n* = 5), and L‐theanine 1000 mg/kg group (−15–0 min: *n* = 3; 0–30 min: *n* = 4; 30–60 min: *n* = 7; 60–90 min: *n* = 6; 90–120 min: *n* = 3; 120–150 min: *n* = 4; 150–180 min: *n* = 7).

### 3.3 Urinary L‐Theanine Concentration During OGTT

**FIGURE 3:**
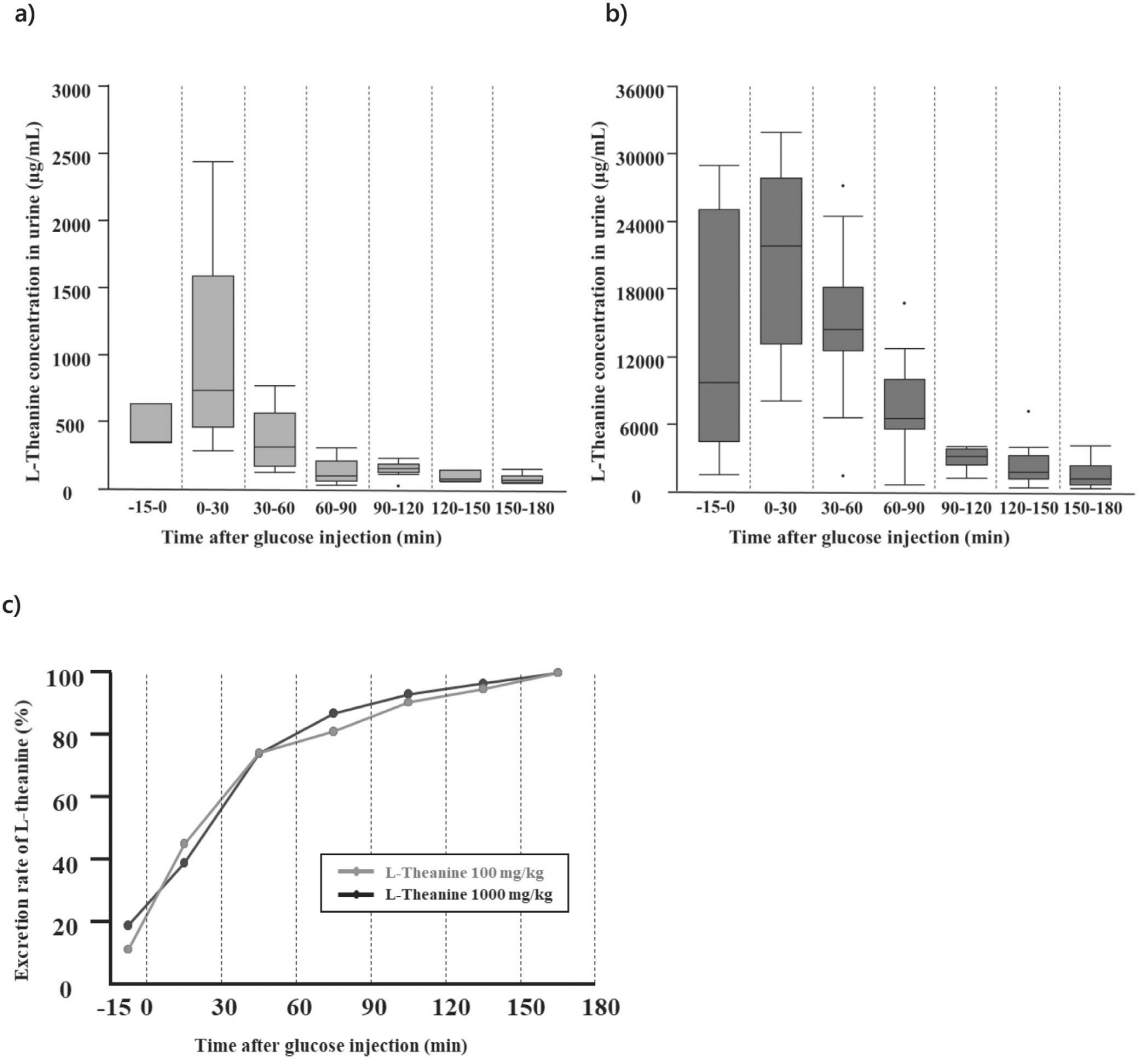
Urinary L‐theanine concentrations during OGTT. (a) and (b) show the urinary excretion of L‐theanine over 180 min following oral administration of 100 mg/kg and 1000 mg/kg, respectively. Values are displayed using box‐and‐whisker plots, wherein the box represents the interquartile range (25th–75th percentiles) and the median is indicated using a line within the box. Outliers beyond the typical range are plotted as individual dots. (c) displays the cumulative excretion rate at each time point, expressed as a percentage of the total amount excreted over the 180‐min period. Sample sizes for each group were as follows: L‐theanine 100 mg/kg group (−15–0 min: *N* = 3; 0–30 min: *N* = 8; 30–60 min: *N* = 14; 60–90 min: *N* = 7; 90–120 min: *N* = 11; 120–150 min: *N* = 6; 150–180 min: *N* = 6) and L‐theanine 1000 mg/kg group (−15–0 min: *N* = 5; 0–30 min: *N* = 9; 30–60 min: *N* = 16; 60–90 min: *N* = 12; 90–120 min: *N* = 7; 120–150 min: *N* = 12; 150–180 min: *N* = 9).

The urinary L‐theanine concentrations are shown in Figures 3a,b and Table 4, and the percentage of L‐theanine excretion at each time point, normalized to a total excretion of 100% over 180 min, is presented in Figure 3c. Urinary L‐theanine concentrations in both the 100 mg/kg and 1000 mg/kg groups reached their maximum in the 0–30 min urine. The median peak concentration in the 1000 mg/kg group was approximately thirty times higher than that in the 100 mg/kg group. The percentage of L‐theanine excretion at each hour relative to total excretion over 180 min was similar in both groups, 74% up to 60 min after glucose administration in both groups, and remained similar in the subsequent excretion rates.

**TABLE 4:** Urinary L‐theanine concentrations at each time point during the OGTT.

Group	−15–0	0–30	30–60	60–90	90–120	120–150	150–180
L‐Theanine 100 mg/kg	346.6 [345.1–632.4]	733.0 [462.2–1586.0]	313.8 [168.5–566.4]	92.3 [60.8–204.2]	151.8 [118.7–181.6]	68.9 [57.7–137.4]	63.9 [45.0–94.9]
L‐Theanine 1000 mg/kg	9716.4 [4467.0–25091.5]	21881.4 [13212.1–27870.0]	14394.5 [12557.2–18184.1]	6569.2 [5611.6–10036.2]	3134.2 [2455.0–3806.9]	1735.7 [1149.9–3230.6]	1198.6 [640.0–2383.9]

*Note:* L‐theanine 100 mg/kg group (−15–0 min: *n* = 3; 0–30 min: *n* = 8; 30–60 min: *n* = 14; 60–90 min: *n* = 7; 90–120 min: *n* = 11; 120–150 min: *n* = 6; 150–180 min: *n* = 6) and L‐theanine 1000 mg/kg group (−15–0 min: *n* = 5; 0–30 min: *n* = 9; 30–60 min: *n* = 16; 60–90 min: *n* = 12; 90–120 min: *n* = 7; 120–150 min: *n* = 12; 150–180 min: *n* = 9).

### 3.4 Urinary Concentration of Amino Acids Other Than L‐Theanine Over 180 Min

**FIGURE 4:**
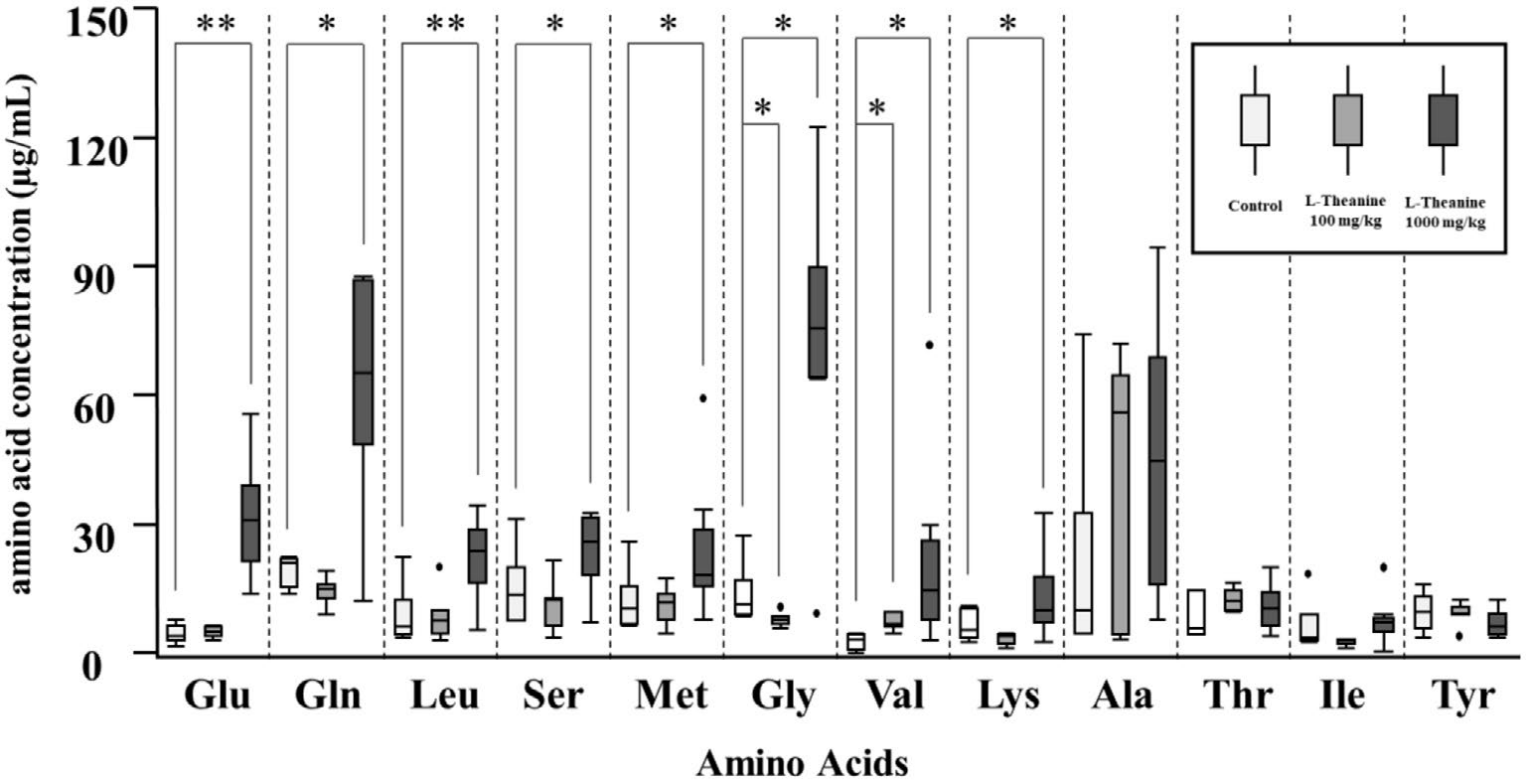
Urinary concentrations of amino acids other than L‐theanine over 180 min. Values are displayed using box‐and‐whisker plots, wherein the box represents the interquartile range (25th–75th percentiles) and the median is indicated using a line within the box. Outliers beyond the typical range are plotted as individual dots. Sample sizes for each group were as follows: Control group (Glu: *N* = 9; Gln: *N* = 10; Leu: *N* = 10; Ser: *N* = 9; Met: *N* = 10; Gly: *N* = 8; Val: *N* = 9; Lys: *N* = 10; Ala: *N* = 10; Thr: *N* = 9; Ile: *N* = 9; Tyr: *N* = 7); L‐theanine 100 mg/kg group (Glu: *N* = 6; Gln: *N* = 6; Leu: *N* = 6; Ser: *N* = 6; Met: *N* = 6; Gly: *N* = 6; Val: *N* = 4; Lys: *N* = 6; Ala: *N* = 6; Thr: *N* = 3; Ile: *N* = 6; Tyr: *N* = 6); L‐theanine 1000 mg/kg group (Glu: *N* = 7; Gln: *N* = 7; Leu: *N* = 7; Ser: *N* = 7; Met: *N* = 7; Gly: *N* = 7; Val: *N* = 7; Lys: *N* = 7; Ala: *N* = 7; Thr: *N* = 6; Ile: *N* = 7; Tyr: *N* = 7). Statistically significant differences are indicated by asterisks (*p < 0.05, **p < 0.01).

The urinary concentrations of glutamic acid (Glu), glutamine (Gln), leucine (Leu), serine (Ser), methionine (Met), glycine (Gly), valine (Val), lysine (Lys), alanine (Ala), threonine (Thr), isoleucine (Ile), and tyrosine (Tyr) were measured. The urinary concentrations of these amino acids are shown in Figure 4 and Table 5. In the group administered 100 mg/kg of L‐theanine, there were no significant differences in the concentrations of these amino acids compared to the control group. However, in the group administered 1000 mg/kg of L‐theanine, the urinary concentrations of Glu, Gln, Leu, Ser, Met, Gly, Val, and Lys were significantly higher than in the control group and increased approximately 2 to 10 times compared to the control group. On the other hand, the concentrations of Ala, Thr, Ile, and Tyr showed no significant differences among any of the groups.

**TABLE 5:** Amino acid concentrations in whole urine collected 180 min after the start of the OGTT.

Group	Glu	Gln	Leu	Ser	Met	Gly	Val	Lys	Ala	Thr	Ile	Tyr
Control	4.0 [2.9–6.3]	21.1 [15.3–22.1]	6.2 [4.2–12.4]	13.5 [7.5–20.0]	10.3 [6.7–15.5]	11.4 [8.8–17.0]	3.1 [0.7–4.3]	5.5 [3.5–10.2]	9.9 [4.8–32.9]	5.8 [4.4–14.6]	3.5 [2.8–8.8]	9.6 [5.8–13.1]
L‐Theanine 100 mg/kg	5.1 [4.1–6.0]	15.0 [12.7–15.9]	7.7 [4.8–10.0]	12.4 [6.5–13.0]	11.7 [7.9–13.9]	7.9 [7.0–8.4]	6.8 [6.0–9.6]	4.1 [2.3–4.2]	56.2 [4.4–64.5]	12.0 [10.0–14.5]	3.0 [2.1–3.2]	9.3 [8.8–10.6]
L‐Theanine 1000 mg/kg	30.8 [21.4–39.2]	65.4 [48.5–86.9]	23.7 [16.5–28.7]	25.8 [18.2–31.7]	18.3 [15.8–28.7]	75.5 [64.1–89.9]	14.5 [7.7–26.3]	10.0 [7.0–17.9]	44.8 [15.9–68.7]	10.4 [6.3–14.1]	7.3 [5.0–8.3]	6.3 [4.2–9.4]

*Note:* Control group (Glu: *n* = 9; Gln: *n* = 10; Leu: *n* = 10; Ser: *n* = 9; Met: *n* = 10; Gly: *n* = 8; Val: *n* = 9; Lys: *n* = 10; Ala: *n* = 10; Thr: *n* = 9; Ile: *n* = 9; Tyr: *n* = 7); L‐theanine 100 mg/kg group (Glu: *n* = 6; Gln: *n* = 6; Leu: *n* = 6; Ser: *n* = 6; Met: *n* = 6; Gly: *n* = 6; Val: *n* = 4; Lys: *n* = 6; Ala: *n* = 6; Thr: *n* = 3; Ile: *n* = 6; Tyr: *n* = 6); L‐theanine 1000 mg/kg group (Glu: *n* = 7; Gln: *n* = 7; Leu: *n* = 7; Ser: *n* = 7; Met: *n* = 7; Gly: *n* = 7; Val: *n* = 7; Lys: *n* = 7; Ala: *n* = 7; Thr: *n* = 6; Ile: *n* = 7; Tyr: *n* = 7).

### 3.5 Urinary Sodium Concentration During OGTT

**FIGURE 5:**
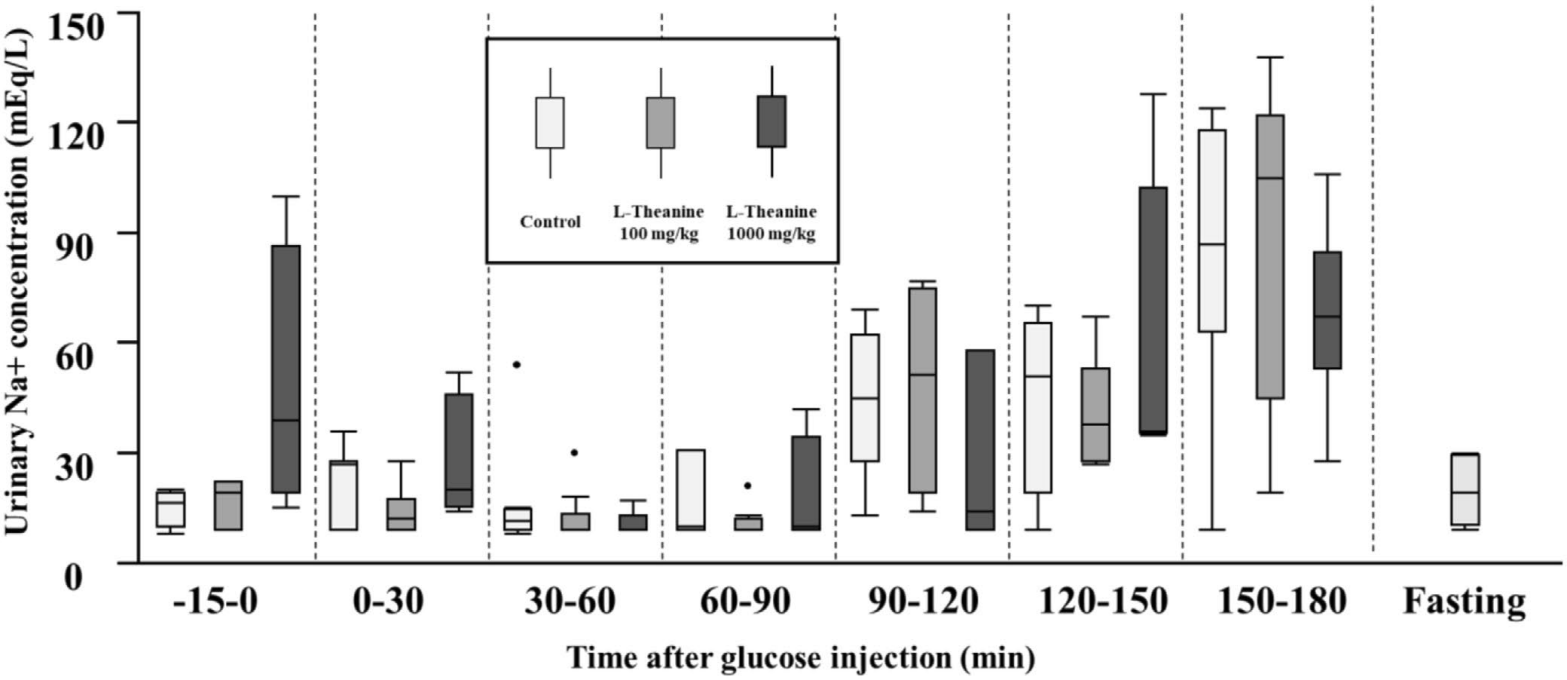
Urinary sodium concentrations during OGTT. Values are displayed using box‐and‐whisker plots, wherein the box represents the interquartile range (25th–75th percentiles) and the median is indicated using a line within the box. Outliers beyond the typical range are plotted as individual dots. Sample sizes for each group were as follows: Control group (−15–0 min: *N* = 4; 0–30 min: *N* = 7; 30–60 min: *N* = 8; 60–90 min: *N* = 7; 90–120 min: *N* = 5; 120–150 min: *N* = 4; 150–180 min: *N* = 7); L‐theanine 100 mg/kg group (−15–0 min: *N* = 3; 0–30 min: *N* = 6; 30–60 min: *N* = 9; 60–90 min: *N* = 8; 90–120 min: *N* = 4; 120–150 min: *N* = 5; 150–180 min: *N* = 6); L‐theanine 1000 mg/kg group (−15–0 min: *N* = 4; 0–30 min: *N* = 5; 30–60 min: *N* = 7; 60–90 min: *N* = 6; 90–120 min: *N* = 3; 120–150 min: *N* = 5; 150–180 min: *N* = 7). Statistically significant differences are indicated by asterisks (*p < 0.05, **p < 0.01).

The urinary sodium concentration (Na+) during OGTT is shown in Figure 5 and Table 6. The Na + concentration in the control group was approximately 20 mEq/L in the random urine collected during fasting. After the start of the OGTT, the Na + concentration fluctuated between approximately 15 and 22 mEq/L during the periods of −15 to 0, 0–30, 30–60, and 60–90 min. After 90 min, the concentration increased, reaching approximately 45 mEq/L during the 90–120 min period and 120–150 min, and 85 mEq/L during 150–180 min. In the group administered 100 mg/kg, no significant difference was noted in urinary Na + concentration compared to the control group, and the trend was similar. In the group administered 1000 mg/kg, the Na + concentration at −15 to 0 min, immediately after theanine administration, was higher than that of the control group and the 100 mg/kg group. Nevertheless, in the urine during the 90–120 min period was the lowest, at less than 27 mEq/L. Although no significant difference was observed, the increase in urinary Na + concentration was delayed, with an increase being observed after 120 min. In the urine collected during the 150–180 min period, the group administered 1000 mg/kg showed the lowest Na + concentration, approximately 68 mEq/L.

**TABLE 6:** Sodium concentrations at each time point during the OGTT.

Group	−15–0	0–30	30–60	60–90	90–120	120–150	150–180	Fasting
Control	16.5 [10.0–19.3]	27.0 [9.0–28.0]	11.5 [9.0–14.8]	10.0 [9.0–31.0]	45.0 [28.0–62.5]	51.0 [19.3–65.5]	87.0 [63.0–118.0]	19.0 [10.5–29.5]
L‐Theanine 100 mg/kg	19.0 [9.0–22.0]	12.0 [9.0–17.5]	9.0 [9.0–13.5]	9.0 [9.0–12.0]	51.5 [19.3–74.8]	38.0 [28.0–53.0]	105.0 [45.0–122.0]	
L‐Theanine 1000 mg/kg	39.0 [19.3–86.5]	20.0 [15.5–46.0]	9.0 [9.0–13.0]	10.0 [9.0–34.5]	14.0 [9.0–58.0]	36.0 [35.5–102.5]	67.0 [53.0–85.0]	

*Note:* Control group (−15–0 min: *n* = 4; 0–30 min: *n* = 7; 30–60 min: *n* = 8; 60–90 min: *n* = 7; 90–120 min: *n* = 5; 120–150 min: *n* = 4; 150–180 min: *n* = 7); L‐theanine 100 mg/kg group (−15–0 min: *n* = 3; 0–30 min: *n* = 6; 30–60 min: *n* = 9; 60–90 min: *n* = 8; 90–120 min: *n* = 4; 120–150 min: *n* = 5; 150–180 min: *n* = 6); L‐theanine 1000 mg/kg group (−15–0 min: *n* = 4; 0–30 min: *n* = 5; 30–60 min: *n* = 7; 60–90 min: *n* = 6; 90–120 min: *n* = 3; 120–150 min: *n* = 5; 150–180 min: *n* = 7). Fasting group (*n* = 5).

## 4 Discussion

**FIGURE 6:**
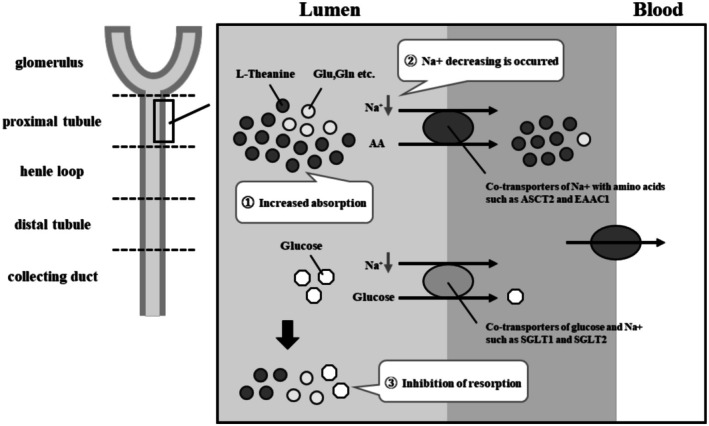
Renal tubular transporters involved in glucose and amino acid excretion and reabsorption: Schematic overview and possible suppressive effects by L‐theanine.

Although the present study is limited by a relatively small sample size, L‐theanine doses being substantially higher than those of typical human intake, and the effects assessed only during an oral glucose tolerance test, it is the first to report the potential of L‐theanine as a supplement that can improve postprandial hyperglycemia. In addition, although only two doses, 100 and 1000 mg/kg, were examined, the evaluation of these two doses, which were expected to produce a pronounced difference, allowed us to gain insights into the sites of action of L‐theanine. We found that at 1000 mg/kg, L‐theanine reduced the increase in blood glucose levels after glucose administration and increased urinary excretion of glucose, Glu, Gln, Leu, Ser, Met, Gly, Val, and Lys compared to that of the control and 100 mg/kg L‐theanine groups. These results, which suppressed the rise in blood glucose levels and increased the excretion of amino acids such as Glu into the urine, recall the article by Kano et al. that reports the relationship between body weight and amino acid transporters. Kano et al. reported that mice in which genes for chaperones such as “Collectrin” that form amino acid transporters such as BAT1 were knocked out were able to reduce weight gain (Kano et al. 2024). Our results suggest that L‐theanine may be reabsorbed by the same transporter that reabsorbs Gln and others in the renal proximal tubules. In addition, the OGTT results in the group administered 1000 mg/kg L‐theanine showed that the amount of Na + excreted in the urine was continuously reduced compared to the other groups, suggesting that L‐theanine may be reabsorbed by amino acid transporter driven by Na+ (Avissar et al. 2001; Hediger et al. 2013; Kanai et al. 2013; Pizzagalli et al. 2021; Porton et al. 2013; Shayakul et al. 1997; Verrey et al. 2009). It was hypothesized that when a large amount of L‐theanine is present in the renal tubules, such as when administered at a dose of 1000 mg/kg, it would reduce the Na + concentration in the proximal tubules and indirectly inhibit Na + –driven glucose transporters (SGLTs), thereby causing large amounts of glucose to be excreted in the urine (Figure 6) (Wright 2001; Wright et al. 2011).

Our new findings allow us to provide an in‐depth discussion of the papers reported so far. A report by Yan et al., who studied the effects of L‐theanine on blood glucose levels and insulin secretion, revealed that L‐theanine reduced serum glucose and insulin concentrations in rats (Yan et al. 2017). In contrast to the findings of Yan et al., our study did not show a decrease in blood insulin levels, likely because our data were obtained from an OGTT. However, it is possible that the enhanced urinary glucose excretion led to a sustained reduction in blood glucose levels, which may ultimately explain the results observed by Yan et al. Peng et al. also reported that L‐theanine improved weight gain and insulin sensitivity in mice fed a high‐fat diet by regulating AMPK in adipocytes (Peng et al. 2021). Combining our results with those of Kano et al. suggests that the suppression of weight gain and improvement of insulin sensitivity may be the result of a decrease in the efficiency of sugar utilization involving amino acid transporters (Kano et al. 2024). Based on the above, the fact that L‐theanine causes more glucose to be excreted in the urine and suppresses the rise in blood glucose levels may be involved in energy metabolism, suppressing weight gain and improving hyperglycemic conditions.

This study revealed that L‐theanine enhances urinary glucose excretion to the extent that it affects the rise in OGTT blood glucose, but the mechanism of this effect could not be clarified. Moreover, in this study, the urinary excretion rate of L‐theanine did not differ significantly depending on the administered dose. Additionally, considering that the amino acids whose concentrations were increased in the group administered 1000 mg/kg L‐theanine were Glu, Gln, Leu, Ser, Met, Gly, Val, and Lys, it was suggested that although L‐theanine is reabsorbed by amino acid transporters such as ASCT2 and EAAC1, which use the amino acids whose excretion was increased as transport substrates, the specificity of the transporter is not high (Avissar et al. 2001; Pizzagalli et al. 2021; Porton et al. 2013; Shayakul et al. 1997). If L‐theanine had high specificity for transporters such as ASCT2 and EAAC1, it would be expected that at a dose of 100 mg/kg, L‐theanine is reabsorbed more efficiently than at a dose of 1000 mg/kg, and the excretion rate is lower at 100 mg/kg. Furthermore, in the case of 100 mg/kg administration, it is conceivable that the reabsorption of L‐theanine would be prioritized over that of other neutral amino acids such as Gln, thereby promoting the urinary excretion of those amino acids. However, as these possibilities were not supported by our results, it is suggested that L‐theanine may not have high specificity for these transporters compared to other neutral amino acids. It is suggested that L‐theanine may be reabsorbed in the kidney via a certain type of amino acid transporter in a sodium (Na^+^)‐dependent manner, thereby suppressing glucose reabsorption. Furthermore, Clemmensen et al. reported that the administration of 1000 mg/kg Arg to mice lowers blood glucose levels; however, this effect was attributed to enhanced secretion of insulin and glucagon‐like peptide 1, without investigating glucose excretion via the kidneys (Clemmensen et al. 2013). Notably, we found no reports indicating that high intake of Gln or Gly, which were affected by excretion following theanine administration in this study, influences blood glucose levels. As L‐theanine is not a proteinogenic amino acid and is not incorporated into energy metabolism, it may promote urinary glucose excretion without being metabolized, potentially leading to reduced blood glucose levels and body weight reduction.

Finally, it is important to consider the relative strength of the hypoglycemic effect of L‐theanine. Administration of L‐theanine at 1000 mg/kg in mice resulted in urinary glucose concentrations exceeding 1000 mg/dL; however, based on the report by Lee et al. regarding dapagliflozin, this effect appears to be substantially weaker than that of an antidiabetic agent (Lee et al. 2018). Lee et al. reported the urinary glucose excretion following administration of the SGLT2 inhibitor dapagliflozin at a dose of 1 mg/kg in C57BL/6 mice. According to their findings, during an OGTT similar to ours, administration of dapagliflozin at 1 mg/kg led to urinary glucose excretion of 10.7 mg/g body weight over approximately 24 h. When comparing our 3‐h excretion data and the rate of glucose excretion per unit time, dapagliflozin at 1 mg/kg induced approximately eightfold greater urinary glucose excretion than L‐theanine at 1000 mg/kg. In light of these considerations, our findings suggest that while L‐theanine is unlikely to serve as a therapeutic agent for diabetes, it may have the potential as a supplement that can improve postprandial hyperglycemia. Because L‐theanine is an amino acid found in tea that people are familiar with, it may be more readily accepted by people who wish to prevent diabetes. While daily tea consumption may contribute to the treatment of diabetes, examining green tea intake to accurately monitor the course of diabetes treatment may also be necessary. Therefore, it will be important to further elucidate the mechanism by which L‐theanine excretes glucose into the urine.

## 5 Conclusions

L‐theanine suppresses the increase in blood glucose levels occurring during an OGTT in mice by promoting glucose excretion in the urine. While further investigation is needed to clarify the underlying mechanism, our study findings suggest that L‐theanine has the function of suppressing a rise in blood glucose levels and has the potential to contribute to the prevention of diabetes.

## Author Contributions

Material preparation and data collection and analysis were performed by Shinnosuke Yamaura and Koki Sadamori. The first draft of the manuscript was written by Koji Komori. Kou Kawada and Nobuyuki Kuramoto commented on previous versions of the manuscript. Reiko Konishi, Takashi Majima, and Akira Mukai were involved in the instructional discussions or critical review of important intellectual content. Kyosuke Uno and Koichi Kawada were involved in the analysis and interpretation of the data and critical review of the paper. All authors have read and approved the final version of the manuscript.

## Disclosure

Statements and declarations: We declare that while writing this paper, we have used a generative AI tool such as ChatGPT to check certain English expressions but have not used it for logical progression or composition.

## Ethics Statement

This study was approved by the Institutional Animal Care Committee of Setsunan University (K22‐27 and K23‐26).

## Conflicts of Interest

The authors declare no conflicts of interest.

## Data Availability

All the data acquired is included in this article.
